# Proteomic Analysis of Breast Cancer Resistance to the Anticancer Drug RH1 Reveals the Importance of Cancer Stem Cells

**DOI:** 10.3390/cancers11070972

**Published:** 2019-07-11

**Authors:** Dalius Kuciauskas, Nadezda Dreize, Marija Ger, Algirdas Kaupinis, Kristijonas Zemaitis, Vaidotas Stankevicius, Kestutis Suziedelis, Jonas Cicenas, Lee M. Graves, Mindaugas Valius

**Affiliations:** 1 Proteomics Center, Institute of Biochemistry, Vilnius University Life Sciences Center, Vilnius University, 10223 Vilnius, Lithuania; 2 Laboratory of Molecular Oncology, National Cancer Institute, 08660 Vilnius, Lithuania; 3 MAP Kinase Resource, 3027 Bern, Switzerland; 4 Department of Pharmacology, University of North Carolina at Chapel Hill, Chapel Hill, NC 27599, USA

**Keywords:** RH1, chemotherapy, cancer drug resistance, JNK, c-KIT, protein kinases, label-free proteomics, MIBs, phosphoproteome, cancer stem cells

## Abstract

Antitumor drug resistance remains a major challenge in cancer chemotherapy. Here we investigated the mechanism of acquired resistance to a novel anticancer agent RH1 designed to be activated in cancer cells by the NQO1 enzyme. Data show that in some cancer cells RH1 may act in an NQO1-independent way. Differential proteomic analysis of breast cancer cells with acquired resistance to RH1 revealed changes in cell energy, amino acid metabolism and G2/M cell cycle transition regulation. Analysis of phosphoproteomics and protein kinase activity by multiplexed kinase inhibitor beads showed an increase in the activity of protein kinases involved in the cell cycle and stemness regulation and downregulation of proapoptotic kinases such as JNK in RH1-resistant cells. Suppression of JNK leads to the increase of cancer cell resistance to RH1. Moreover, resistant cells have enhanced expression of stem cell factor (SCF) and stem cell markers. Inhibition of SCF receptor c-KIT resulted in the attenuation of cancer stem cell enrichment and decreased amounts of tumor-initiating cells. RH1-resistant cells also acquire resistance to conventional therapeutics while remaining susceptible to c-KIT-targeted therapy. Data show that RH1 can be useful to treat cancers in the NQO1-independent way, and targeting of the cancer stem cells might be an effective approach for combating resistance to RH1 therapy.

## 1. Introduction

Acquired tumor resistance often limits the efficiency of current chemotherapeutic cancer treatments. The acquired resistance arises during treatment through various therapy-induced adaptive responses due to intrinsic tumor heterogeneity [1]. Identification of biological processes and pathways that are crucial for the development of drug resistance provides potential molecular targets for successful combinatorial treatment.

Multiple studies show that chemotherapy leads to the accumulation of cancer stem cells (CSC) that are responsible for tumor initiation and repopulation after the treatment [2,3,4]. However, there is no consensus on what role do CSCs play in acquired drug resistance [2]; moreover, some studies avoid a CSC concept focusing instead on the intrinsic tumor heterogeneity and cell survival-promoting features [5]. Other studies, on the contrary, demonstrate the existence of distinct populations of CSCs potentially representing different disease subtypes in the same tumor that may require different therapies [6]. There are no universal and widely acknowledged protein markers or biological features for CSC definition. Most of the research, however, heavily relies on the usage of specific markers presented in CSCs, including CD133, CD44, ALDH, and ABCG2 [7], while other associate stemness with phenotype changes, for instance, the epithelial to mesenchymal transition (EMT) [8]. Nevertheless, the selective targeting of cells, which exhibit so-called CSC properties, is a developing strategy for cancer treatment that currently undergoes multiple clinical trials including advanced breast, ovarian and small-cell lung cancers [9,10].

A promising strategy to attack selectively cancer cells is based on targeting distinct cancer cell properties. One of these cancer cell-specific drugs is 2,5-diaziridinyl-3-(hydroxymethyl)-6-methyl-1,4-benzoquinone (RH1), a novel aziridinyl benzoquinone-based cytotoxic compound that has shown promising results as an antitumor agent during phase I trials and has been recommended for the phase II trials [11]. RH1 was created as a prodrug designed to be activated by the two-electron reductase NAD(P)H:quinone oxidoreductase (NQO1) which is expressed at high levels in the most human solid tumors [12]. In addition, one-electron reductases, such as NADPH cytochrome P450 reductase also have been shown to play some role in RH1 conversion into the more active form in cancer cells [13]. Therefore, after the two-electron reduction of RH1 by NQO1 it preferentially induces DNA alkylation and cross-linking [14], while reduction by non-NQO1 reductases ultimately results in the generation of free radicals, oxidative stress and, consequentially, cytotoxicity in cancer cells [13,15].

It is widely acknowledged that virtually any anticancer drug can lead to the development of drug resistance. Cancer cell resistance to RH1 in the experimental models is usually linked to the downregulation of RH1-activating enzymes. However, as we showed previously that besides NQO1 downregulation multiple other biological processes, such as cell cycle, DNA repair, energy production and metabolism proteins can also contribute to the acquired RH1 resistance [16].

Here, we demonstrate that anticancer drug RH1 was effective against triple-negative breast cancer cells. Remarkably, we show that these cells do not possess any detectable NQO1 catalytic activity demonstrating that RH1 conveys its cytotoxicity by a new mechanism(s). Moreover, our high content data of the global differential proteome, kinome and phosphoproteome analysis point to the RH1 resistance mechanisms as changes in cell cycle progression, cell signaling, as well as the increase in cancer stem cell phenotype. Validation of these changes shows that the major mechanism defining acquired RH1 resistance of the triple negative breast cancer cells is linked to the alteration of the activity of multiple protein kinases including downregulation of JNK and enrichment of cancer stem cell population via autocrine stimulation of stem cell factor (SCF) and c-KIT signaling axis.

## 2. Results and Discussion

### 2.1. Establishment and Characterization of RH1-Resistant MDA-MB-231 Cells

**Figure 1:**
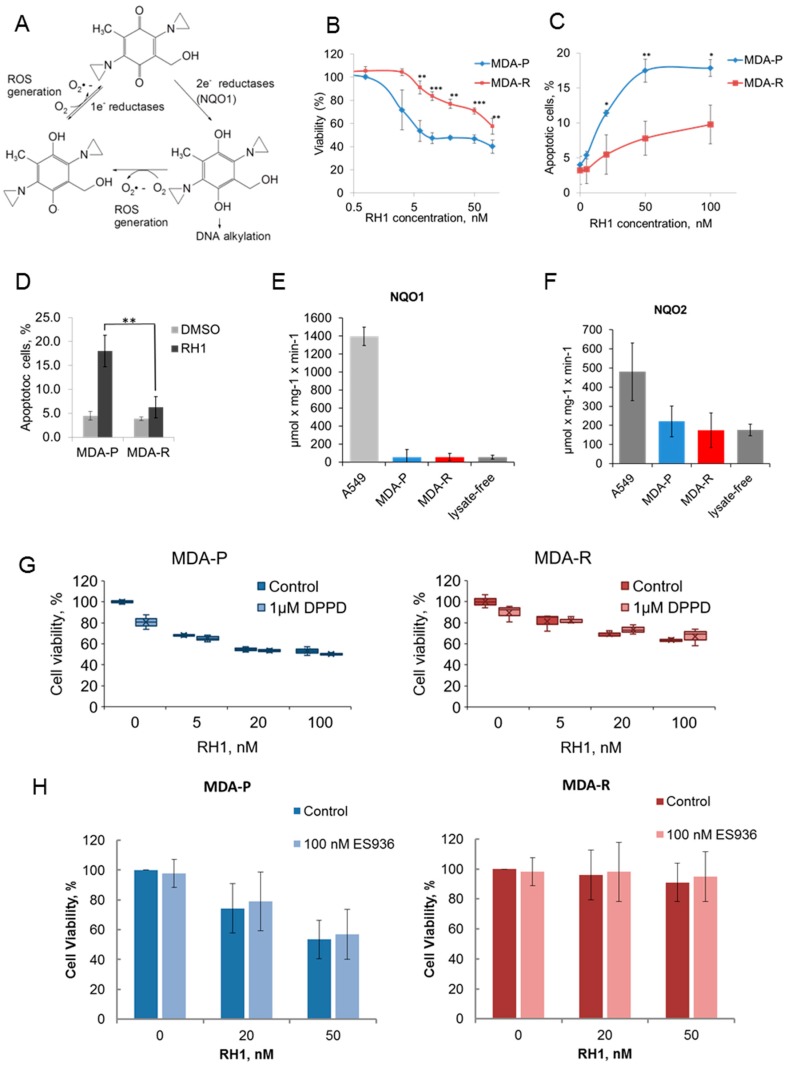
Characterization of RH1 resistant cells. (**A**). Molecular mechanism of the prodrug RH1 bioreduction. (**B**). Viability test. MDA-P and MDA-R cells were treated with increasing concentrations of RH1 for 2 h. Cell survival after 96 h was estimated by MTT assay. Bars are ± SD, significant differences are marked by asterisks: ** *p* < 0.05, *** *p* < 0.01, *t*-test, *n* = 3. (**C**). Apoptosis assay. MDA-P and MDA-R cells were exposed to increasing concentrations of RH1 for 2 h and then cultured in drug-free medium for another 48 h. Apoptosis was assayed using acridine orange/ethidium bromide staining. Bars are ± SD, significant differences are marked by asterisks: * *p* < 0.1, ** *p* < 0.05, *t*-test, *n* = 3. (**D**). Quantification of flow cytometric nexin-based apoptosis assay. Cells were untreated or treated with 50 nM RH1 for 2 h and stained with Guava Nexin reagent after 48 h. Bars are ± SD, significant difference is marked by asterisks: ** *p* < 0.05, *t*-test, *n* = 3. (**E**). NQO1 activity assay. The initial rate of RH1 reduction using NADPH cofactor represents NQO1 activity in the cell lysates where cell lysate of A549 cells is acting as a positive control. Bars are ± SD, differences between A549 as a positive control and other samples are significant (*p* < 0.01), *n* = 3. (**F**). NQO2 activity assay. The initial rate of RH1 reduction using NMEH cofactor represents NQO2 activity in the cell lysates. Bars are ± SD, difference between A549 as a positive control and other samples are significant (*p* < 0.01), *n* = 3. (**G**). MDA-P and MDA-R were treated with RH1 in the presence or absence of antioxidant DPPD. Cell survival after 96 h was estimated by MTT assay. Bars are ± SD. (**H**). MDA-P and MDA-R were treated with RH1 in the presence or absence of NQO1 inhibitor ES936. Cell survival after 96 h was estimated by MTT assay. Bars are ± SD.

RH1 has been designed as a pro-drug to be activated mainly by NQO1 overexpressed in many solid tumors [17] as well as one-electron reductases, such as NADPH cytochrome P450 reductase, which has been shown to play a minor role on RH1 conversion into the active form [18] (Figure 1A). Acquired cancer cell resistance to RH1 is usually caused in part by the decreased level of RH1-reducing (i.e., RH1 activating) enzymes [16,19]. However, the experimental data show that RH1 can be effective in treating cancer cells that do not have elevated NQO1 activity, such as triple-negative breast cancer cells MDA-MB-231 or other cancer cell lines [13,20]. To open a wider avenue of RH1 usage in cancer treatment of tumor cells that do not possess elevated NQO1 activity, here we have investigated sensitivity to RH1 as well as mechanisms of acquired resistance to RH1, independent of the drug-reducing enzymes.

RH1-resistant MDA-MB-231 cells were derived from the parental drug-sensitive cell line by continuous selection with RH1. Cells were treated with the increasing dose of RH1 with subsequent recovery and repopulation. The IC_50_ values have been measured for MDA-MB-231 parental (designated thereafter as MDA-P) and RH1 selected MDA-MB-231 (designated thereafter as MDA-R) cells by the MTT test (Figure 1B). In MDA-R cells, the IC_50_ concentration of RH1 was 91.9 ± 9.7 nM compared to 6.5 ± 1.8 nM in the original MDA-P line, showing a 14-fold increase of RH1 resistance.

Next, we tested the RH1 ability to induce apoptosis in both cell lines by applying two different biological tests. Apoptosis induction by RH1 was measured using acridine orange/ethidium bromide staining (Figure 1C). The significant difference in the RH1-induced apoptotic cell death was observed at concentrations of RH1 reaching IC_50_ concentration for MDA-R cells. Additionally, annexin-based apoptosis assay demonstrated that RH1 causes apoptotic cell death and confirmed that MDA-R were more resistant to RH1-induced apoptosis compared to MDA-P (Figure 1D). It is widely accepted that NQO1 contributes most to the RH1 activation by its reduction. As previously shown, NQO2 can also catalyze two- and four-electron reduction reactions on quinones [21] and is potential RH1 bioactivating enzyme [12]. To test whether any residual NQO1 or NQO2 activity in MDA-P or MDA-R contributes to RH1 reduction, we measured NQO1 and NQO2 enzyme substrate consumption kinetic rates in cell lysates. A549 cell line known for high NQO1 activity contributing to RH1 toxicity [22] was used as a positive control. Our data show that both cell lines demonstrate no observable NQO1 activity (Figure 1E) and there is no statistically significant difference between NOQ2 activity in MDA-P and MDA-R cells (Figure 1F).

Since RH1 reduction potentially can generate ROS that might contribute to the drug cytotoxicity, we tested whether the treatment of cells with ROS scavengers prevents RH1 from killing breast cancer cells. Data show that treatment of cells with DPPD (Figure 1G) or NAC (data not shown) does not compromise RH1 toxicity. Besides, NQO1 inhibitor ES936 did not affect RH1-dependent cell death either in MDA-P or in MDA-R cells (Figure 1H) supporting the hypothesis that RH1 kills MDA-MB-231 cells independently of NQO1 catalytic activity. Taken together, data show that in the triple negative breast cancer cells RH1 affects cell viability in the NQO1-independent way.

### 2.2. Differential Global Proteomics of Breast Cancer Cells Predicts Cellular Mechanisms of RH1 Resistance

To examine changes in proteome associated with resistance to RH1 we performed high-throughput differential label-free quantitative proteomic analysis of MDA-P and MDA-R cells using high-definition mass spectrometry (HDMS) technology. MDA-P and MDA-R cells were left untreated or treated with 20 nM of RH1 for 2 h, cell lysates were prepared after 4 h, to assess early RH1-induced proteome changes; or 16 h, to evaluate events prior to the onset of apoptosis induction.

We have performed three biological experiments, whereby ~5500 proteins in total in RH1-sensitive and -resistant MDA-MB-231 cell proteomes have been identified and quantified (Supplementary Table S1).

**Figure 2:**
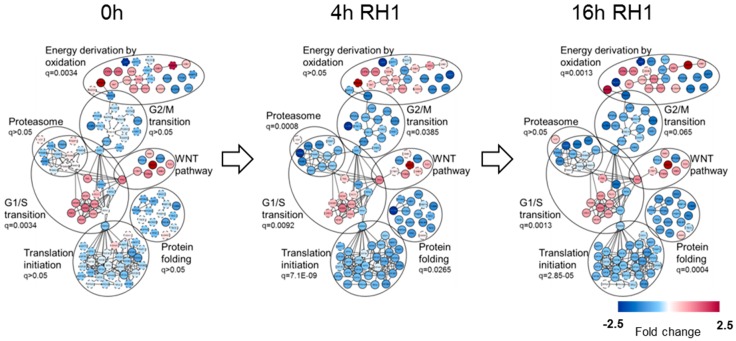
Functional analysis of global proteome dynamic changes between MDA-P and MDA-R cells. MDA-P and MDA-R cells, untreated or treated with 20 nM RH1 for 2 h followed by 2 or 14 h recovery period (4 and 16 h from the beginning of treatment respectively), were subjected to in-depth quantitative proteomic analysis. Protein differential expression, interaction and functional annotation network was built by means of Cytoscape software with GeneMANIA application. The main functional clusters with enrichment q-value are represented. The color of the nodes demonstrates protein level change in RH1-resistant MDA-R cells.

Protein-protein interaction network of differentially expressed proteins for each treatment time was built in Cytoscape [23] using GeneMANIA plugin [24] and fold change in protein levels was depicted in color gradient. The main functional clusters comprising proteins identified by GO term enrichment analysis are depicted in Figure 2 and listed in Supplementary Table S2.

The protein set from RH1-untreated cells shows a significant enrichment of GO terms related to DNA replication and G1/S transition of mitotic cell cycle, ribosome biogenesis and linked to mitochondrial matrix and energy production by oxidation. In addition, among the proteins elevated in MDA-R cells we pinpointed a population of genes that participate in WNT signaling pathway.

The 4 h and 16 h of treatment with RH1 of MDA-R cells compared to MDA-P revealed massive decrease in translation initiation proteins, protein folding-regulating species, G2/M cell cycle transition, as well as proteasome complex-related proteins in the RH1-resistant cells. Initial differences in the level of energy metabolism-related proteins, G1/S transition and WNT pathway proteins between MDA-P and MDA-R cells remained unchanged.

Taken together, functional analysis of global proteome differences between RH1-sensitive and resistant cell lines highlighted biological processes potentially responsible for the development of acquired RH1 resistance. The change in basic level of cell energy metabolism and mitochondrial proteins, most of which were increased in MDA-R, might be linked to the drug resistance through quinone-based compound detoxification and enhanced antioxidant defense. One-carbon metabolic enzymes involved in serine and the folate cycle SHTM2 and MTHFD1L elevated in MDA-R cells are also responsible for the synthesis of NADPH and nucleotides. These enzymes are known to be crucial for the growth and survival of cancer cells [25]. Additionally, diminishing or loss of SHTM2 and MTHFD1L are associated with sensitizing of cancer cells to oxidant-induced cell death [26].

On the other hand, G1/S cell cycle transition-regulating proteins are mostly upregulated, and G2/M phase proteins are downregulated in RH1-resistant cells. The differential expression becomes more pronounced after the short-term treatment with RH1. The change in cell cycle progression between MDA-P and MDA-R and perturbation caused by RH1 was further confirmed by the cell cycle assay, which shows that RH1 causes cell cycle arrest in G2/M phase in MDA-P but not in resistant MDA-R cells (Supplementary Figure S3).

Analysis of enriched signaling pathways showed the elevated level of WNT signaling proteins including the key downstream component of WNT canonical pathway β-catenin (CTNNB1). It is known that Wnt/β-catenin signaling pathway plays a significant role in triple negative breast cancer development and progression and is considered an important potential therapeutic target [27]. Importantly, Wnt signaling has also been found to be essential for the propagation of cancer stem cells [28]. In conjunction, data show that proteasome complex components were also downregulated in MDA-R cells; low proteasome activity is known to be a potential functional marker for CSC and treatment resistance [29].

In summary, global differential proteome analysis of MDA-P and MDA-R cells shows that changes in the cell energy and amino acid metabolism, as well as cell cycle and the increase in CSC-like properties might be responsible for the acquisition of resistance to RH1.

### 2.3. Phosphoproteome and Kinome of RH1 Resistant Breast Cancer Cells Highlight Signaling Pathways Responsible for the RH1 Resistance

First, we applied the multiplexed kinase inhibitor bead (MIB) approach described previously [30] to examine differences in the kinomes between a RH1-sensitive and RH1-resistant cell lines. Kinases were isolated from MDA-P and MDA-R cell lysates using MIB enrichment, which captures a broad range of protein kinases by five different inhibitors covalently coupled to beads. Bead-immobilized inhibitors compete with ATP binding in the active kinases, thus only activated protein kinases can associate with MIB and later be identified and quantitated by mass spectrometry. Therefore, this assay shows the total amount of active protein kinases, as well as kinase-interacting proteins in the cell lysate [31,32,33,34,35,36,37].

We identified a total of 693 proteins, representing each protein kinase group and several non-protein kinases involved in the regulation of various biological processes. To visualize the trend in kinase abundance changes between MDA-R/MDA-P cells we set a cutoff of ±1.5-fold; this threshold was based on previous analysis of technical replicates [30], using guidelines proposed by Unwin, et al. [38]. The expression of 42 activated protein kinases was increased in MDA-R cells as compared to MDA-P and the activity of 52 kinases was decreased (Supplementary Table S3).

**Figure 3:**
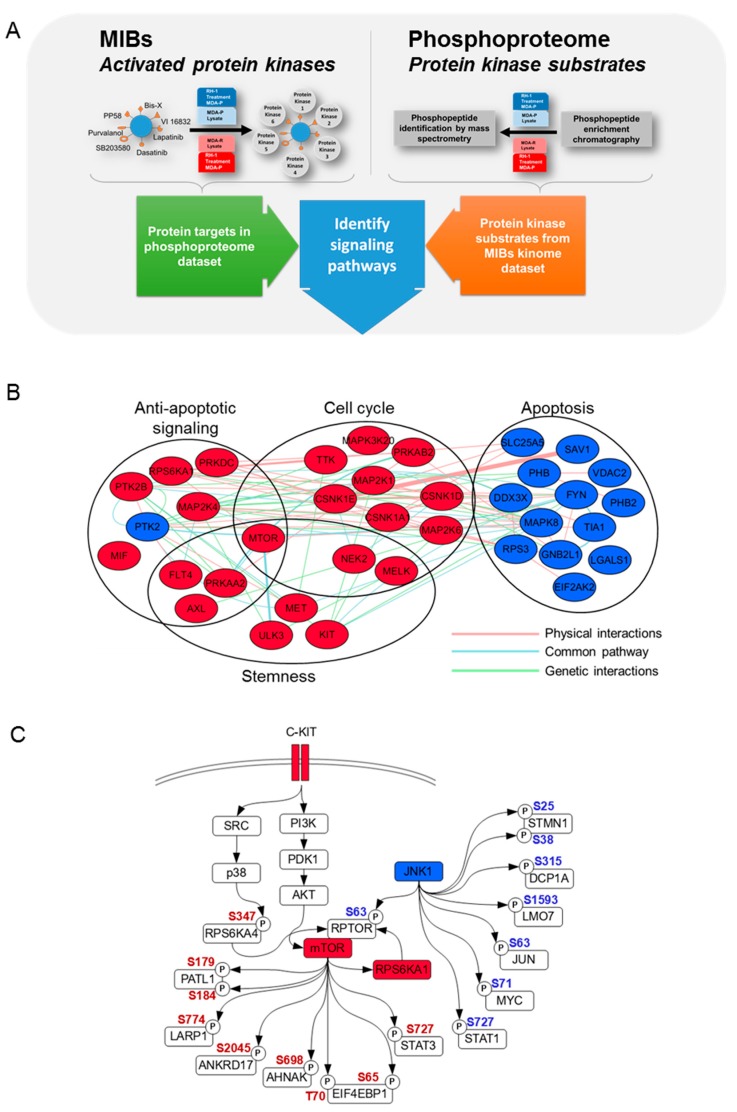
Protein phosphorylation highlights signaling pathways in RH1-resistant cells. (**A**). Experimental workflow for kinome and phosphoproteome analysis. (**B**). MIB analysis of increased and decreased kinase activity between MDA-P and MDA-R cells. MDA-P and MDA-R cells were subjected to MIB analysis, followed by quantitative proteomic analysis. Kinase differential activity, interaction and functional annotation network was built by means of Cytoscape software with GeneMANIA application. The main functional clusters are represented and the color of the nodes demonstrates kinase activity change (red—increased, blue—decreased). (**C**). JNK1 and c-KIT-AKT-mTOR signaling pathways. Most biologically important pathways were analyzed combining MIB and phosphoproteome datasets. Red node color indicates increased activity of MIB dataset kinases, blue—decreased. Red phosphosite color indicates increased phosphorylation in the phosphoproteome dataset, blue—decreased. AKT and JNK phosphosites were identified by Western blot. Nodes without color indicate proteins that belong to the pathway but have neither been identified nor their phosphorylation changes were observed in either of datasets.

We used bioinformatics and biocuration to annotate the biological functions of these kinases and to estimate which functions could be important for the resistance of MDA-R cells (Figure 3A). Data reveal the increase of activated kinases, such as RPS6KA1, MTOR, TTK, CSNK1D, CSNK1A1, MELK, CSNK1E, PRKAB2, PRKAA2, MAP2K1, MAP3K20, MAP2K6, NEK2, and PRKDC confirming our previous observation that status of the cell cycle is important for the RH1-resistant cells (Figure 3B). Cell cycle analysis indicated that RH1 caused cell cycle arrest at the G2/M phase boundary and most of the RH1-resistant cells tend to bypass RH1-induced cell cycle arrest (Supplementary Figure S1).

On the other hand, we observe the diminution of activity of kinases that contribute to apoptosis, such as JNK1, PHB, SAV1, LGALS1, VDAC2, SLC25A5, FYN, TIA1, PHB2, RPS3, EIF2AK2, DDX3X and RACK1. Then again, negative regulators of apoptosis, such as RPS6KA1, AXL, MAP2K4, PRKAA2, PRKDC, MTOR, FLT4 and PTK2B have increased activity. All of that illustrates the diminished apoptosis in RH1-resistant cells.

In addition, the increase of the activity of such kinase as mTOR, AXL, ULK3, MET, MELK, PRKAA2, NEK2 and FLT4 confirms our previous observation, that RH1-resistant cells are prone to stemness, since all of these kinases are known to be involved in cancer stem-like cell maintenance [39,40,41,42,43,44,45,46,47].

Taken together, these results suggest that there are several biological processes which are changed in the resistant cells and contribute to the resistance to RH1. Since the acquisition of drug resistance is often evolved through simultaneous dysregulation of multiple biological pathways [48] we show that cell cycle bypass, negative regulation of apoptosis and stem-like cell maintenance are the most important of those processes in the RH1-resistant cells. Notable it is also a fact, that some of kinases with increased activity are involved in broad regulation of those processes, for example mTOR being involved in all of them (Figure 3B).

As a second part to investigate the importance of protein phosphorylation, we compared phosphoproteome of parental cell line to that of resistant cells using phosphopeptide enrichment technique combined with quantitative mass spectrometry. This assay produced a vast amount of information, thus, based on our previous data, we concentrated our efforts on JNK pathway as well as c-KIT and mTOR and applied bioinformatics and curation analysis to identify the proteins phosphorylated in these pathways (Figure 3C).

**Figure 4:**
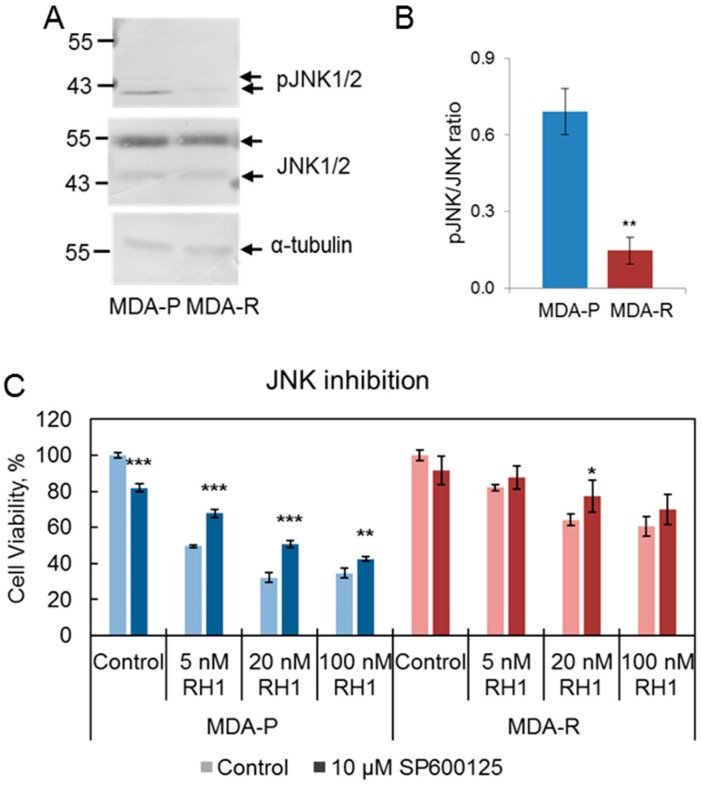
The decrease in JNK activation is related to RH1 resistance. (**A**). Western blot analysis showing the level of pJNK (Thr183/Tyr185) in MDA-P and MDA-R cells. α-tubulin is shown as loading control. (**B**). Densitometric analysis of pJNK and JNK western blot. Bars are ± SD, significant difference is marked by asterisks: ** *p* < 0.05, *t*-test, *n* = 4. (**C**). Viability test after JNK inhibition. MDA-P and MDA-R cells were pre-treated with JNK inhibitor SP600125 for 1 h and then treated with increasing concentrations of RH1 for 2 h. Cell survival after 96 h was estimated by MTT assay. Bars are ± SD, significant differences are marked by asterisks: * *p* < 0.1, ** *p* < 0.05, *** *p* < 0.01, ANOVA, *n* = 3.

Initially we have observed that JNK1 activity is decreased in the MDA-R cells according to the MIBs data; therefore, we searched JNK target phosphopeptides using the GPS phosphorylation prediction program, as well as PhosphoSitePlus resource data [49]. Eight phosphosites belonging to seven proteins that could be phosphorylated by JNK kinase were identified. This includes mTOR interaction partner RPTOR, and the prominent JNK targets JUN and STAT1, all of which are involved in the induction of apoptosis [50,51,52], cell cycle arrest [53,54,55] and transcription control [56,57,58]. Other JNK targets are less phosphorylated in the drug-resistant cells, including LMO7 involved in ubiquitination [59], DCP1A in mRNA decapping [60] and STMN1 that contributes to microtubule depolymerization [61]. Moreover, the decrease in JNK activating phosphosite (Thr183 and Tyr185) in RH1 resistant cell lines was validated by western blot analysis (Figure 4A).

On the other hand, the c-KIT signaling pathway contains at least three members which are increased in the MIB dataset, namely c-KIT tyrosine kinase itself, m-TOR and RPS6KA1 serine/threonine kinases. We also identified peptides of seven proteins from the phosphoproteome dataset, which can be phosphorylated by mTOR and have increased phosphorylation in the resistant cells (Figure 3C; Supplementary Table S4).

Remarkably, EIF4EBP1 and ANKRD17 proteins are involved in G1/S transition of the mitotic cell cycle [62,63]. In addition, c-KIT-mTOR axis that might lead to phosphorylation and activation of STAT3, which is involved in the negative regulation of apoptosis [64], positive regulation of cell cycle [65] and transcription [66], and, most importantly, in regulation of the cancer cells stemness [67]. Similarly, mTOR kinase apparently phosphorylates LARP1 which is involved in the regulation of cell proliferation [68], negative regulation of translation [69], positive regulation of macroautophagy [70] and chemoresistance [71]. Another mTOR potential phosphorylation targets are PATL1, which controls cytoplasmic mRNA processing body assembly [72] and thus mRNA deadenylation and decapping [73], and AHNAK that regulates protein oligomerization and RNA splicing [74].

Since neither MIBs nor phosphoproteome experimental datasets revealed a direct link between c-KIT and mTOR activation, thus, we decided to analyze phosphorylation of the most prominent mTOR upstream kinase AKT activity. Immunoblot analysis of AKT phosphorylation in both MDA parental and resistant cell lines indicated increased phosphorylation on Thr308 and Ser473 (Supplementary Figure S2). Moreover, AKT target phosphopeptides belonging to six proteins were identified in the phosphoproteome dataset. Notably, AKT phosphorylation target CDK2 is known as a key cell cycle regulator, controlling both G1/S and G2/M transitions [75]. We also identified an increase an in β-catenin (CTNBB1) phosphorylation leading to the augmented transcriptional activity and maintenance of cancer cell stem-like properties, such as tumor initiation, proliferation and invasion [76,77]. Moreover, other AKT targets that could be important for cancer cell resistance were identified, such as IRS1, the regulator of cell proliferation and invasion [78], and HSPB1, which can function as apoptosis regulator [79]. Finally, another possible AKT targets are HTT, involved in vesicle trafficking [80], and FLNC, acting as a scaffold protein [81], although, their contribution to the drug resistance remains elusive.

The increase in phosphorylation of Ser347 of RPS6KA4 has been detected in the phosphoproteome dataset. RPS6KA4 is a known p38 binding partner and substrate [82]. However, no significant upregulation of basal p38 phosphorylation was confirmed by western blot (data not shown) suggesting that the increase in p38-dependent phosphorylation of RPS6KA4 was caused by either very dynamically phosphorylated-dephosphorylated p38 or its specific subcellular-localized fraction.

Therefore, phosphoproteome analysis illustrates the importance of AKT-mediated phosphorylation as well as highlight activated c-KIT-AKT-mTOR axis in cells bearing RH1 resistant phenotype.

Altogether combined data of two complementary proteomic techniques, kinome analysis using MIBs and phosphoproteome analysis by phosphopeptide enrichment reveal the alterations of proteins involved in the regulation of cell cycle, apoptosis and transcription, as well as of the cell stemness modulation. These data pinpoint the decrease of JNK activity as an important event which downregulates apoptosis, and highlight the activation of c-KIT receptor and downstream proteins as potential cell survival and stemness regulators.

In addition, our recent data based on computer modeling of RH1 molecular docking with ATP binding domain of various protein kinases reveals that RH1 might compete with ATP binding and therefore can inhibit kinase catalytic activity. It is wrth noting that c-KIT is among the most susceptible protein kinases in silico concurrently inhibited by RH1 [83], once again highlighting c-KIT’s role in RH1 resistance.

### 2.4. Decrease in JNK Activity Mediates Resistance to RH1

Since kinome and phosphoproteome experiments show the downregulation of JNK activity, we assayed JNK phosphorylation on Thr183 and Tyr185 in MDA-P and MDA-R cell lines by western blot (Figure 4A,B). The observed decrease in JNK phosphorylation in MDA-R cells compared to MDA-P cells confirmed the lower JNK activity in the resistant cells.

Next, we evaluated the role of JNK activity diminution in the cell resistance to RH1. JNK inhibition with SP600125 resulted in partial improvement of RH1 resistance of MDA-P cells. The sensitivity to RH1 of MDA-R cells was affected to a lesser extent or not affected at all (Figure 4C). This shows that the decrease of JNK activity contributes to the RH1 resistance confirming the importance of JNK family kinases in RH1-mediated apoptosis [84].

### 2.5. Cancer Stem Cell Population Is Enriched in the Breast Cancer Cells Resistant to RH1

Acquired cancer drug resistance is linked to the expansion of cancer stem cell subpopulation [2,3,4]. Moreover, our OMICS data consistently point to the increase of stem cells in the drug-resistant cell culture. Therefore, we analyzed how stem-cell specific features manifest in RH1 resistant cell line.

**Figure 5:**
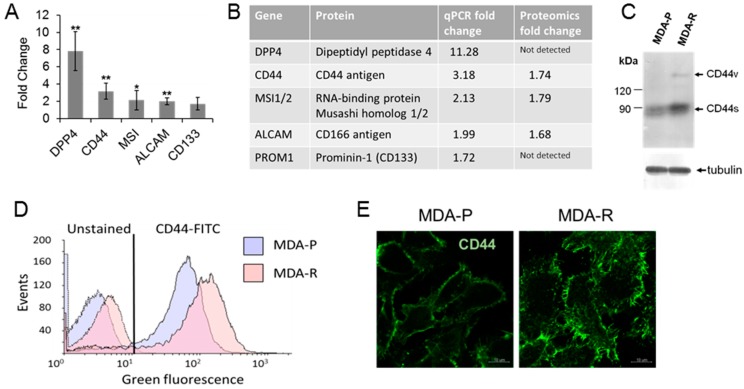
RH1 resistant cells show an increased level of CSC markers. (**A**). Relative expression of selected CSC markers measured by RT-qPCR in MDA-R compared to MDA-P. The results are the mean of 3 independent experiments; bars are ± SD (ANOVA; * *p* < 0.05; ** *p* < 0.01). (**B**). Comparison of CSC markers detected by RT-qPCR with quantitative proteomic analysis results. (**C**). Western blot analysis of various isoforms of CD44 expression in MDA-P and MDA-R cell lines. (**D**). Flow cytometry aggregated data graph showing autofluorescence and CD44 expression in MDA-P and MDA-R cells. Data combined from unstained and CD44-FITC antibody-stained samples. E. Representative comparative confocal images of CD44 expression in MDA-P and MDA-R cells. Scale bar: 10 µm.

First, a set of known cancer stem cell biomarkers was analyzed by means of qRT-PCR: DPP4, CD44, MSI1, ALCAM and CD133 (Figure 5A). The expression of all the biomarkers with exception for CD133 was significantly increased in MDA-R line. The increase of CD44, MSI1/2 and ALCAM mRNA amount correlates with their protein level observed in the global proteome. Although, DPP4 and CD133 in the proteomic experiments were not detected (Figure 5B), we have confirmed DPP4 higher expression by the confocal microscopy (data not shown).

Next, to validate proteomic and RT-PCR data we assayed CD44 expression by flow cytometry, western blot and confocal microscopy. CD44 is well known marker for the identification of CSCs in breast cancer, and cells bearing CD44^+^/CD24^−^ phenotype exhibit properties of cancer stem cells and high tumor initiating capacity [85]. Western blot analysis shows the increase in CD44 level in RH1 resistant cell line (Figure 5C). It also shows the increased expression of CD44 variant isoform formed by alternative splicing. Different variant isoforms of CD44 can be associated to different breast cancer subtypes and clinical markers [86]. Flow cytometric analysis confirms that MDA-R cells possess subpopulation with higher CD44 levels than MDA-P cells (Figure 5D). Moreover, unstained RH1-resistant cells exhibit elevated green autofluorescence (Figure 5D), which is also emerging as novel indicator for cell stemness that correlates with a wide variety of proofed CSC markers expression in vitro and in vivo [87]. Finally, we have examined CD44 expression and distribution in MDA-P and MDA-R cells by indirect immunofluorescence staining. Images in Figure 5E illustrate strong membrane CD44 immunostaining in MDA-R cells compared to MDA-P.

Taken together, these data demonstrate that RH1-resistant cells exhibit elevated levels of multiple CSC markers. Therefore, acquired breast cancer cells resistance to RH1 can be at least partially explained by the enrichment of CSC drug-resistant population.

### 2.6. Stem Cell Factor Receptor c-KIT Contributes to the Acquisition of RH1 Resistance in Breast Cancer Cells

**Figure 6:**
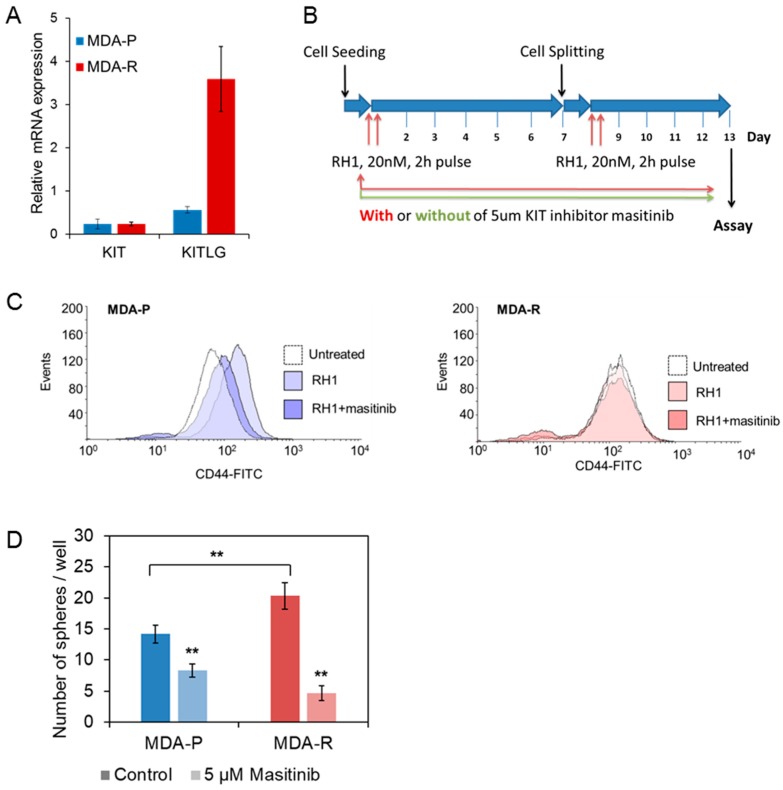
KIT inhibition attenuates MDA-P selection toward CSC induced by the double treatment with RH1. (**A**). Relative expression levels of KIT receptor and its ligand KITLG in MDA-P and MDA-R cells measured by RT-qPCR. The results are the mean of 3 independent experiments; bars are ± SD (ANOVA; * *p* < 0.05; ** *p* < 0.01). (**B**). Experimental time chart of cells short-time selection without and with KIT inhibition. (**C**). MDA-P (blue) or MDA-R (red) cells were pulse exposed or not exposed twice to 20 nM RH1 with or without of continuous treatment with KIT receptor inhibitor masitinib (5 µm). After 13 days cells were stained with CD44-FITC antibody and analyzed by flow cytometry. (**D**). MDA-P and MDA-R cells were subjected to sphere forming assay with or without 5µM masitinib treatment. Spheres were stained with MTT dye and counted using ImageJ software. The results are the mean of 3 independent experiments, two replicates each; bars are ± SD (ANOVA; * *p* <0.05; ** *p* < 0.01).

CSC phenotype in RH1 resistant cancer cell line could arise from activation of known CSC marker c-KIT [88] as indicates our OMICS data. The assessment of growth factor and its ligand expression with qRT-PCR revealed elevated expression of stem cell factor (SCF), which is the ligand of c-KIT receptor (Figure 6A). Although the amount of c-KIT receptor mRNA does not change, significantly, the MIBs assay shows the increase of c-KIT kinase activity in MDA-R cells (Figure 3C, Supplementary Table S3). These data show that the MDA-R cells very likely have enhanced autocrine-stimulated c-KIT.

Since CD44^high^ breast cancer phenotype correlates positively with c-KIT expression [89], we investigated how the prolonged treatment with c-KIT inhibitor masitinib affects the CD44^high^ population in MDA-P and MDA-cells. Cells were exposed to the pulses of 20 nM of sub-lethal RH1 concentration and left to recover in the growth media with or without masitinib for six days. The treatment was repeated twice resulting in 13 days-long short-term selection (timeline depicted in Figure 6B), therefore, allowing us to recapture a short-term selection.

The level of CD44 in MDA-P and MDA-R cells after this short-time selection was evaluated using flow cytometry (Figure 6C). The treatment with RH1 led to the selection of MDA-P cells with an increased level of CD44. However, when RH1 treatment was combined with the treatment of nonlethal dose of c-KIT inhibitor the increase of CD44^high^ CSCs was dramatically reduced. No changes in the CD44^high^ population during this short-term selection assay was observed in MDA-R cells that already are resistant to the drug. This might be explained that previous long-term treatment of cells with the drug, which has led to the RH1-resistant cell line generation, cannot produce more CD44^high^ cells during this short-time selection experiment.

Thus, data show that c-KIT inhibitor when applied at the nonlethal concentration diminishes MDA-P cell selection by RH1 showing the importance of SCF—c-KIT loop in the CSC-like cells generation. One of the major characteristics of breast cancer stem-like cells is the ability to initiate tumor and form mammospheres in vitro as demonstrated with highly malignant CD44^+^/CD24^−^ cells [90]. To evaluate tumor-initiating capacity of RH1 resistant cell line and to elucidate the role of c-KIT activation in the maintenance of stem-like state, we performed the cell sphere-forming assay. MDA-P and MDA-R cells were grown in a non-adherent matrix containing a limited resource of growth factors that resembles similar tumor formation conditions in vivo. Under those circumstances, MDA-R cells formed significantly more spheres than MDA-P cells showing the increased capability of CSC-enriched drug-resistant cell line to initiate tumor formation. Importantly, treatment of cells with c-KIT inhibitor masitinib had more detrimental effect on MDA-R sphere forming capability than on MDA-P (Figure 6D).

In summary, MDA-R cells are characterized by the elevated SCF expression, and the activated c-KIT receptor promotes the accumulation of CD44^high^ cell population in drug-sensitive MDA-P cells. In conjunction with the proteomic data, which show that RH1-resistant cells have more activated c-KIT and have more activated stemness-related pathways, results strongly suggest that SCF-c-KIT autocrine regulation axis plays a crucial role in acquiring RH1 resistance through the enrichment of CSC population. Combining RH1 treatment with c-KIT inhibitors such as masitinib might prevent or significantly reduce the development of CSCs-based acquired resistance to RH1 in breast cancer therapy.

### 2.7. c-KIT Is a Potential Target in the Therapy of RH1-Resistant Breast Cancer Cells

Conventional chemotherapy drugs, such as docetaxel, 5-fluorouracil, oxaliplatin and epirubicin, were tested as potential second-line therapy candidates to treat RH1-resistant cells. All these drugs trigger cell death by a different mechanism and have been used in various breast cancer treatment regimens. Fluorouracil, epirubicin and docetaxel are used as adjuvant treatment for women with node-positive early breast cancer [91,92], similarly, platinum-based chemotherapy achieves increased response rates for triple-negative breast cancer tumors [93].

**Figure 7:**
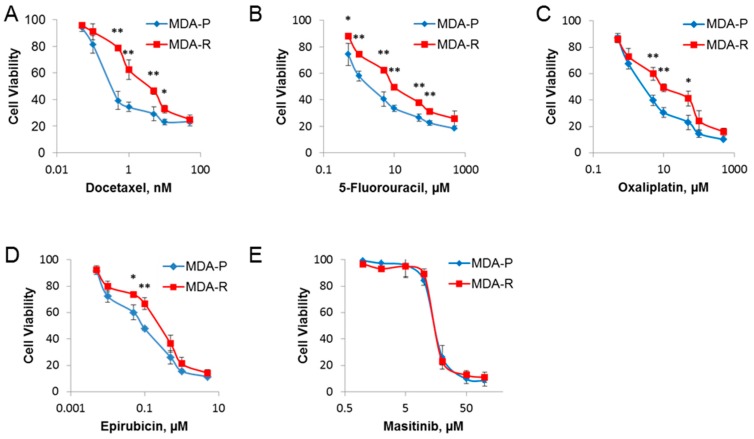
RH1 resistant cells acquire insusceptibility to conventional chemotherapy drugs, but not to c-KIT-targeted therapy. MDA-P and MDA-R cells were treated with increasing concentrations of drugs for 48 h: (**A**) docetaxel; (**B**) 5-fluorouracil; (**C**) oxaliplatin; (**D**) epirubicin; or for 72 h for (**E**) masitinib. Cell viability was measured after 96 h using the MTT assay. The results are the mean of 3 independent experiments; bars are ± SD (ANOVA; * *p* < 0.05; ** *p* < 0.01).

Data show that RH1-resistant cells also acquired multidrug resistance to the numerous conventional therapeutics. The highest change in IC_50_ values established by viability test was for docetaxel (9-fold increase, from 0.33 ± 0.10 nM in MDA-P to 2.96 ± 0.74 nM in MDA-R); the efficiency of other conventional drugs was also compromised in RH1-resistant cells (Figure 7A–D).

Receptor tyrosine kinase inhibitors or neutralizing antibodies are potential combination therapy drugs. For example, trastuzumab in HER2-positive breast cancer [94] or imatinib mesylate (Gleevec) in chronic myelogenous leukemia [95,96] are widely used for clinical treatment. Remarkably, our data show that while MDA-R manifests elevated resistance to the conventional chemotherapy drugs its dose-dependent sensitivity to masitinib does not change compared to parental MDA-P line in the cell viability assay (Figure 7E). However, when cells were tested for tumor initiation capabilities by sphere formation assay in vitro, MDA-R cells turned to be more sensitive to the masitinib treatment (Figure 6D) at the nonlethal c-KIT inhibitor doses. Also, it is notable that masitinib at the lower concentration (5 µM) can prevent MDA-P selection into more CSC-like cells during the short-term treatment assay (Figure 6C). This indicates the dual role of c-KIT inhibitor treatment: initially, masitinib at the lower concentration inhibits generation of CSC-like cells during RH1 selection; later, at the higher dose the inhibitor of c-KIT acting on-target and/or off-target effectively kills RH1-sensitive as well as RH1-resistant cells.

Our data clearly show that conventional chemotherapy drugs, such as docetaxel, 5-fluorouracil, oxaliplatin and epirubicin, should not be recommended as second-line drugs due to the acquisition of tolerance to those substances in the RH1-resistant breast cancer cells. On the other hand, targeted therapy via RTK inhibition, such as masitinib treatment may be successfully used to destroy both the primary and RH1-resistant cells as well as to prevent tumor cells accumulate CSC-like species.

Data reveal an important role for the SCF-c-KIT signaling axis in self-renewal and proliferation of CSCs, and they suggest that SCF-c-KIT signaling blockade could improve the antitumor efficacy of chemotherapy as it has been demonstrated with human NSCLC [97].

Notably, since masitinib is already in the trials for ovarian (NCT02490488), pancreatic (NCT00789633) cancer, gastro-intestinal stromal tumors (NCT00998751, NCT00812240, NCT01506336, NCT02009423, NCT01694277), metastatic melanoma (NCT01280565), as well as second-line therapy for metastatic colorectal cancer (NCT02605044), it makes RH1 and masitinib a promising combination in cancer treatment.

## 3. Materials and Methods

### 3.1. Cell Culture, Drug Treatment and Establishment of RH1-Resistant Cell Lines

Human breast adenocarcinoma MDA-MB-231 cells (obtained from ECACC, Salisbury, UK) and A549 (obtained from Cell Lines Service, Eppelheim, Germany) cells were maintained in Dulbecco’s modified Eagle’s medium (DMEM) (Life Technologies, Carlsbad, CA, USA) supplemented with 10% fetal bovine serum (FBS) (Life Technologies, Carlsbad, CA, USA) and penicillin-streptomycin antibiotics (Life Technologies, Carlsbad, CA, USA). Considering rapid RH1 clearance from the blood during the chemotherapy [11], cells were always treated with RH1 for 2 h. Then media with RH1 was removed, cells washed with PBS and left to recover in fresh growth media. The time of the treatment is counted since the input of RH1.

The RH1-resistant subline was established by treating MDA-MB-231 cells with RH1 (IC_50_, increasing from 2 nM to 35 nM) for 2 h followed by cell recovery stage. This development period was carried out for 6 months and included 15 treatment cycles. The parental MDA-MB-231 subline, that was cultivated without RH1 treatment, was named MDA-P and the established RH1-resistant subline was named MDA-R.

### 3.2. Antibodies and Inhibitors

FITC-conjugated CD44 mouse monoclonal antibody (MEM-85) for flow cytometry experiments, FITC-conjugated mouse IgG2b for isotype control and phospho-AKT (Thr308) were purchased from Thermo Fisher Scientific (Vilnius, Lithuania). CD44 mouse monoclonal antibody (8E2) for western blot and confocal microscopy, JNK, phospho-JNK, AKT and phospho-AKT (Ser473) antibodies were from Cell Signaling Technology (Danvers, MA, USA).

RH1 was provided by N. Cenas lab (Life Sciences Center, Vilnius University). Docetaxel, 5-fluorouracil, oxaliplatin, epirubicin were purchased from Teva Baltics (Vilnius, Lithuania). Masitinib, ES936 and SP600125 were purchased from Selleckchem (Munich, Germany).

### 3.3. Assessment of Cell Viability and Apoptosis

The resistance of MDA-P and MDA-R lines to RH1, conventional chemotherapy drugs or receptor tyrosine kinase inhibitors was evaluated using MTT assay. The day before treatment MDA-P and MDA-R cells were seeded in 96-well flat-bottomed plates (3000 cells/well). Cells were treated for various agent concentrations for the indicated period of time. After treatment, fresh growth media was applied and the MTT test was performed 96 h after the beginning of the treatment. Media with 0.5 mg/mL MTT was added to each well and incubated for 1 h at 37 °C. The formazan product was dissolved by adding 100 µL dimethylsulfoxide to each well, and the plates were read at 550 nm with Varioskan Flash Multimode Reader (Thermo Scientific, Vilnius, Lithuania). Results were normalized according to untreated control and changes between two cell lines were evaluated.

All measurements were performed in quadruplicate and each experiment was repeated at least three times.

Differences of RH1-induced apoptosis in MDA-MB-231 parental and resistant cell lines were evaluated using acridine orange/ethidium bromide or Guava Nexin (Merck Millipore, Darmstadt, Germany) assay. For acridine orange/ethidium bromide assay MDA-P and MDA-R cells were seeded in 24-well flat-bottomed plates (80 × 10^3^ cells/well). The next day the cells were treated with RH1 at various concentrations for 2 h. 48 h after treatment cells were detached with trypsin and stained with 1 µg/ml acridine orange and 1 µg/mL ethidium bromide in PBS for 5 min. The apoptotic changes were measured by flow cytometry (Guava easyCyte 8HT Flow Cytometer, Merck-Millipore. Darmstadt, Germany). Each experiment was repeated at least three times.

For Guava Nexin assay MDA-P and MDA-R cells were seeded in in 22 cm^2^ dishes. The next day the cells were treated with 50 nM RH1 for 2 h. 48 h after treatment cells were detached with trypsin and stained with Guava Nexin reagent according to manufacturer protocol. The apoptotic changes were measured by flow cytometry (BD FACSCanto II, BD Biosciences, San Jose, CA, USA). Each experiment was repeated at least three times.

### 3.4. RH1 Reduction Assay

For studies of RH1 reduction, cells were grown in 22 cm^2^ cell culture dishes. Cells were scraped off and suspended in 500 μL of buffer containing 25mM Tris-HCl (pH 7.4) and 250mM sucrose. Cells were disrupted with an ultrasonic homogenizer (Bandelin Sonopuls HD 2070, Berlin, Germany) and protein concentration was measured with a Coomassie Plus Bradford Assay (Thermo Scientific, Vilnius, Lithuania). Cell lysates were diluted to a concentration of 1 mg/ml with the same buffer which has been used for cell disruption. The RH1 reduction rate was measured in the presence of the NADPH regeneration system or by adding 500 μM NMeH (reduced N-methylnicotinamide, Santa Cruz Biotechnology, Heidelberg, Germany). For the NADPH regeneration system we used 20 μM NADPH, 10 mM glucose-6-phosphate and 10 µ/mL glucose-6-phosphate dehydrogenase. The reactions were followed spectrometrically using a Hitachi 557 UV-Vis spectrometer at 37 °C. The RH1 reduction was monitored at 328 nm with the NADPH regeneration system or at 318 nm (NMeH isosbestic point) when NMeH was used as a cosubstrate. NMeH oxidation was monitored at 380 nm. Spectral data was first processed with R Studio and then statistical analysis was performed with MS Excel software.

### 3.5. Flow Cytometry and Western Blot

For immunofluorescent flow cytometry experiments, 10^5^ cells were detached with Accutase (Thermo Fisher Scientific), washed with PBS containing 1% bovine serum albumin (BSA) and stained with FITC-conjugated CD44 monoclonal antibody (Thermo Fisher Scientific) for 30 min. After staining, cells were placed in PBS containing 1% BSA and analyzed with BD FACSCanto II flow cytometer (BD Biosciences) with 488-nm blue laser and standard FITC 530/30nm bandpass filter.

For western blot analysis, postnuclear lysates were resolved using SDS-PAGE, the proteins were transferred to polyvinylidene difluoride membrane (Bio-Rad, Hercules, CA, USA) and blocked in Blotto (0.9% NaCl, 8 mM Tris HCl, 2 mM Tris, 1% skimmed milk, 0.025% Tween-20, 0.05% NaN3). The membranes were incubated for 2 h with primary antibody (titer 1:1000) and then for 0.5 h with alkaline phosphatase-conjugated secondary mouse antibody (MilliporeSigma, St. Louis, MO, USA). Blots were developed using nitro-blue tetrazolium and 5-bromo-4-chloro-3-indolylphosphate tolidium salt.

### 3.6. Sphere-Forming Assay

For sphere-forming assay, 24-well plate was coated with 1% agarose in PBS to limit cell attachment to the surface. Then suspended cells were embedded in 0.3% agarose matrix in DMEM medium with 0.1% FBS and Insulin-Transferrin-Selenium (ITS, Life Technologies, Carlsbad, CA, USA) to prevent cell clumping and seeded 500 cells per well. The plate was kept in room temperature for 30 minutes allowing agarose matrix to solidified and then cells were treated with DMEM medium with 0.1% FBS and ITS containing 5 µM masitinib.

Cells were grown for two weeks, cell feeding was performed every five days by adding DMEM medium with 0.1% FBS and ITS. Formed spheres were dyed using MTT dye and visualized using ImageJ software. The experiment was performed in duplicates and repeated three times.

### 3.7. Total RNA Isolation and Real-Time qPCR for Evaluation of the mRNA Expression

Total RNA was isolated from approximately 1 × 10^6^ cells using GeneJET RNA Purification Kit (Thermo Fisher Scientific, Vilnius, Lithuania) according to the manufacturer’s instructions. To validate differential gene expression changes, RevertAid RT Kit (Thermo Fisher Scientific, Vilnius, Lithuania) was used for cDNA synthesis according to the manufacturer’s instructions. Briefly, 1 μg of total RNA was added to 20 µL reverse transcription (RT) reaction volume containing 5 µM random hexamer primers, 1 µM of dNTP mix, 20 U RNase inhibitor and 20 U reverse transcriptase. Then the mixture was incubated at 25 °C for 5 min followed by synthesis at 42 °C for 60 min. and terminated by heating at 70 °C for 5 min. RT-qPCR was performed on MasterCycler RealPlex^4^ RT-PCR system (Eppendorf, Hamburg, Germany.) using 2× KAPA SYBR FAST qPCR Master Mix (KAPA BIOSYSTEMS, Boston, MA, USA) according to manufacturer’s instructions. All reactions were performed in a 10 µl reaction volume containing 5 µL 2× KAPA SYBR FAST qPCR Master Mix, 1 µL 5-fold diluted cDNA, 0.2 µL of 10 µM forward and reverse primer mixture and 3.8 µL of nuclease-free water. The reaction conditions were as follows: pre-denaturation at 95 °C for 3 min. followed by amplification of 40 cycles of 3 s at 95 °C and 30 s min at 60 °C. The relative changes in gene expression were evaluated by ∆∆C_t_ method as described previously [98]. For the normalization of the expression data, HPRT1 was used as a reference gene. Primer sequences used in amplification are shown in Table 1.

### 3.8. Cell lysis and Sample Preparation for Mass Spectrometry Analysis

Cells were grown for 24 h in RH1-free growth media, rinsed 3 times with PBS (37°C), and lysed with 0.5 ml of urea/thiourea lysis buffer (7 M urea, 2 M thiourea, 4% CHAPS, 40 mM DTT, Halt Protease Inhibitor Cocktail (Thermo Fisher Scientific, Vilnius, Lithuania). The lysates were sonicated for 1 min at the amplitude of 20% and 0.4 s pulsations on/off cycles (Sonopuls HD 2070, Bandelin, Berlin, Germany). Homogenized lysates were centrifuged at 20,000 g for 15 min at 4 °C, and the supernatants were collected. The lysates then were stored at −86 °C.

Trypsin digestion was done according to a modified filter-aided sample preparation (FASP) protocol as described by Wisniewski et al. [99]. Briefly, proteins were diluted in 8 M urea, and loaded on Amicon Ultra-30 kDa filters. Following two washes with urea, proteins were alkylated with 50 mM iodoacetamide. Filters were washed twice with urea and twice with 50 mM NH_4_HCO_3_. Proteins digested overnight with TPCK Trypsin 20233 (Thermo Scientific, Vilnius, Lithuania). After overnight digestion, peptides were recovered by centrifugation and then two additional washes using 50% CH3CN were combined, acidified, lyophilized, redissolved in 0.1% formic acid and then fractionated off-line.

### 3.9. Multiplexed Inhibitor Bead Affinity Extraction

Cells were lysed on ice in 50 mM HEPES (pH 7.5), 0.5% Triton X-100, 150 mM NaCl, 1 mM EDTA, 1 mM EGTA, 10 mM sodium fluoride, 2.5 mM sodium orthovanadate, 1 mM PMSF and 1% of phosphatase inhibitor cocktail 3 (MilliporeSigma, St. Louis, MO, USA). Lysates were sonicated 1 min on ice and centrifuged at 22,000 G for 15 minutes at 4 °C. The supernatant was collected and syringe-filtered through a 0.2 mm SFCA membrane. The filtered lysate (approximately 1.5 mg of protein per sample) was brought to 1 M NaCl and precleared by incubating lysate with 500 µl Sepharose CL-4B for 15 min and then incubated with 100 µL multiplexed inhibitor-conjugated beads (MIBs) consisting of Sepharose-conjugated Bisindoylmaleimide-X, dasatinib, Purvalanol B, PP58 and VI16832. The MIBs were washed three times with high-salt and low-salt buffers (50 mM HEPES (pH 7.5), 0.5% Triton X-100, 1 mM EDTA, 1 mM EGTA, and 10 mM sodium fluoride, and 1 M NaCl or 150 mM NaCl, respectively). Proteins were eluted from MIBs with elution buffer containing 7 M urea, 2 M thiourea, 4% CHAPS and 40 mM DTT.

### 3.10. Liquid Chromatography and Mass Spectrometry

Peptides were separated off-line on a 200 × 2.1 mm, 5 µm SCX column (300 µm i.d., 15 cm, packed with POROS 10S) using a gradient of 0–60% B over 20 min with a flow rate of 300 µL/min. Solvent A was 5 mM NaH_2_PO_4_, pH 3.0 in 5% acetonitrile. Solvent B was solvent A + 1M NaCl. The separation was monitored at 214 nm and either 2 min five fractions were collected. The fractions were dried using a vacuum centrifuge and resuspended in 30 µL of 0.1% formic acid (FA).

Each of the SCX fractions was analyzed by nano LC-MSE analysis. Peptides were loaded on reversed-phase trap column PST C18, 100 Å, 5 µm, 180 µm × 20 mm (Waters Corporation, Wilmslow, UK) with a flow rate of 15 µL/min using loading buffer of 0.1% formic acid and subsequently separated on HSS-T3 C18 1.8 μm, 75 μm × 250 mm analytical column (Waters Corporation) in 120 min linear gradient (A: 0.1% formic acid, B: 100% CH3CN and 0.1% formic acid) at a flow rate of 300 nL per min; column temperature was kept at 40 °C.

The nano-LC was coupled online through a nano-ESI 7 cm length, 10 mm tip emitter (New Objective, Woburn, MA, USA) with HDMS Synapt G2 mass spectrometer (Waters Corporation). Data were acquired using MassLynx version 4.1 software (Waters Corporation) in positive ion mode. LC-MS data were collected using data-independent acquisition (DIA) mode MSE in combination with online ion mobility separation.

The trap collision energy of mass spectrometer was ramped from 18 to 40 eV for high-energy scans in MSE mode. The trap and transfer collision energy for high-energy scans in HDMS mode were ramped from 4 to 5 eV and from 27 to 50 eV. For both analyses, the mass range was set to 50–2000 Da with a scan time set to 0.9 s. A reference compound [Glu1]-Fibrinopeptide B (Waters Corporation) was infused continuously (500 fmol/µL at a flow rate 500 nL per min) and scanned every 30 s for on-line mass spectrometer calibration purpose. The samples were run in triplicate.

### 3.11. Data Processing, Searching and Analysis

Raw data files were processed and searched using ProteinLynx Global SERVER (PLGS) version 2.5.3 (Waters Corporation). The following parameters were used to generate peak lists: (i) minimum intensity for precursors was set to 150 counts; (ii) minimum intensity for fragment ions was set to 50 counts; (iii) intensity was set to 500 counts. Processed data was analyzed using trypsin as the cleavage protease, one missed cleavage was allowed and fixed modification was set to carbamidomethylation of cysteines, variable modification was set to oxidation of methionine. Minimum identification criteria included 1 fragment ions per peptide, 3 fragment ions per protein and a minimum of 2 peptides per protein. The false discovery rate (FDR) for peptide and protein identification was determined based on the search of a reversed database, which was generated automatically, when global false discovery rate was set to 4%. UniprotKB/SwissProt human database (2015-04-22) was used for protein identification.

Kinome perturbation analysis was performed using TOP3 (3 most intense peptides) quantification data after ISOQuant normalization of raw data sets. Raw data for both experiments was acquired from two experiments each consisting of three technical replicates. Proteins were regarded as altered if difference between mean values of technical replicates was more or equal to 1.5 in any of the two biological experiments. The raw files of datasets are available at ftp://massive.ucsd.edu/MSV000080608.

### 3.12. Computational Functional Analysis of Proteomic Data

For quantitative analysis of global proteome, increase or decrease in protein level of 1.5-fold or more was considered as upregulation or downregulation, respectively. Differential expression analysis was carried out using the Piano R package [100] and false discovery rate (FDR) adjusted p-values (Q-values) were calculated. The protein-protein interaction network of differentially expressed proteins was built using GeneMANIA app (3.4.1) [101] for Cytoscape 3.3.0 [23]. Physical interaction data only was used for network generation, no related genes were added to the network. Gene Ontology (GO) annotations from GeneMANIA were used for GO enrichment analysis of differential proteome, q-value 0.05 and below was considered as significant. Kinome and phosphoproteome biological functions were annotated using a combination of GO enrichment and biocuration (database and literature search).

### 3.13. Phosphoproteome

Cells for phosphoproteome analysis were grown on 15 cm internal diameter dishes to subconfluence and treated with 20 nM RH1 for 2 h followed by 2 h recovery. The cells were lysed with buffer containing 2% SDS, 10 mM Tris/HCl pH 7.6, 0.1 M DTT, protease (Pierce) and phosphatase inhibitors (Sigma Aldrich). A total of 10 dishes were used for a single sample. Lysates were sonicated for 2 min at the amplitude of 15% and 0.4 s pulsations on/off cycles (Sonopuls HD 2070) and heated for 3 min at 95 °C. Homogenized lysates were centrifuged at 20,000 g for 15 min at 4 °C, and the supernatants were collected. Protein concentration was measured using Pierce™ Coomassie Plus (Bradford) Assay Reagent. The lysates then were stored at −86 °C. The peptides were prepared by standard FASP protocol.

Peptide mixture from biological replicates was fractionated by basic reversed-phase (HpH) high-pressure liquid chromatography (HPLC) fractionation using a Waters XBridge BEH130 C18 3.5 μm 4.6 × 250 mm column on a Ultimate 3000 HPLC (Thermo Scientific Dionex, Sunnyvale, CA, USA) operating at 160 µL/min. 10 mM ammonium hydroxide was used as an additive to the mobile phases (B consisted ammonium hydroxide with 90% acetonitrile). Fractionation gradient commenced as follows: 1% B to 45% B in 30 min and ramped to 90% B in 2 min. The gradient was held at 90% B for 5 min before being ramped back to 1% B, where the column was then washed and equilibrated. The number of fractions was set to 10 and later concatenated to 5.

For all experiments, phosphopeptides from each HpH fraction were enriched using titanium dioxide (TiO2) phosphopeptide enrichment kit (Thermo Scientific) following the manufacturer’s protocol.

Data-dependent analysis (DDA) was performed with the nanoAcquity coupled to a Synapt G2 HDMS mass spectrometer (Waters). For DDA, the instrument performed a 0.5 s MS scan (350–1350 scan range) followed by MS/MS acquisition on the top 5 ions with charge state 2+, 3+ and 4+. MS/MS scans range was 50–2000 Da, 0.6 s scan duration with exclusion after 2 MS/MS scans were acquired, and dynamic exclusion of ions within 50mDa of the selected precursor m/z was set to 100 s.

The Progenesis QI for proteomics software (Nonlinear Dynamics, Newcastle upon Tyne, UK) in combination with the Mascot database search tool (2.2.07) was employed to identify and quantify phosphopeptides. The acquired raw-files were imported into the Progenesis software tool for label-free quantification using the default parameters. MS2 spectra were exported directly from Progenesis in mgf format and searched using the MASCOT algorithm. The results were further statically validated by software tool SafeQuant [102]. The software normalizes the identified MS1 peak abundances (extracted ion chromatogram, XIC) across all samples. All quantified phosphopeptides/proteins are assigned an abundance ratio, based on the median XIC. The statistical significance of each ratio is given by its q-value (false discovery rate adjusted *p* values), obtained by calculating modified t-statistic *p* values [103] and adjusting for multiple testing.

## 4. Conclusions

The canonical pathway of RH1 anticancer activity has been attributed to two-electron reduction by NQO1 ultimately inducing DNA alkylation and crosslinking. An additional way of RH1 activation leads to the formation of reactive oxygen species, which is initiated by the single-electron enzymatic reduction of RH1 to semiquinone, subsequently going through redox cycling. However, our data show that neither of those mechanisms are involved in the cytotoxic RH1 action against triple negative breast cancer MDA-MB-231 cells. Moreover, we could establish a MDA-R cell line, which is resistant to RH1 treatment. Proteomics, phosphoproteomics and kinome analysis as well as several kinase activity assessment assays established the importance of diminution JNK activity and c-KIT-AKT-mTOR axis enhancement in the establishment of RH1 resistance in the triple-negative breast cancer cells. Protein kinases and their targets affect several biological functions of the cells, most noteworthily cell cycle, anti-apoptosis as well as cancer stem cell maintenance to cause chemoresistance. Assessment of known cell stemness biomarkers DPP4, CD44, MSI1 and ALCAM as well as autofluorescence showed that resistant cells are enriched with cancer stem cells. Together with OMICS experiments, these data prove that stemness is very important for cell resistance to RH1 treatment. In addition, we found that expression of SCF, the ligand of c-KIT receptor, was elevated in the resistant stem-like cells, which correlated with our discovery that c-KIT activity is also increased. Treatment with masitinib, a tyrosine kinase inhibitor with a preference for c-KIT receptor, shows that the RH1 resistant cell line remained equally sensitive to the masitinib as the parental cells. Moreover, inhibition of SCF-c-KIT signaling prevented enrichment of CSC cells as well as diminished tumor-initiating capacity of the RH1 resistant cells. These results show that c-KIT inhibitors as CSC-targeting agents in combination with RH1 treatment might be used as an effective therapy to treat triple negative breast cancer while overcoming the development of RH1 resistance.

## Figures and Tables

**Table 1:** List of primer sequences used in RT-qPCR.

Gene	Forward Sequence	Reverse Sequence
**DPP4**	5′-AGTGGCACGGCAACACATT-3′	5′-AGAGCTTCTATCCCGATGACTT-3′
**CD44**	5′-CTGCCGCTTTGCAGGTGTA-3′	5′-CATTGTGGGCAAGGTGCTATT-3′
**MSI**	5′-TAAAGTGCTGGCGCAATCG-3′	5′-TCTTCTTCGTTCGAGTCACCA-3′
**ALCAM**	5′-ACTTGACGTACCTCAGAATCTCA-3′	5′-CATCGTCGTACTGCACACTTT-3′
**PROM1**	5′-TTCTTGACCGACTGAGACCCA-3′	5′-TCATGTTCTCCAACGCCTCTT-3′
**KIT**	5′-ACTTGAGGTTTATTCCTGACCCC-3′	5′-GCAGACAGAGCCGATGGTAG-3′
**KITLG**	5′-AATCCTCTCGTCAAAACTGAAGG-3′	5′-CCATCTCGCTTATCCAACAATGA-3′
**HPRT1**	5′-TGCAGACTTTGCTTTCCTTGGTC-3′	5′-CCAACACTTCGTGGGGTCCTT-3′

## Supplementary Materials

The following are available online at https://www.mdpi.com/2072-6694/11/7/972/s1, Table S1: Proteins identified in HDMS analysis, Table S2: Proteins comprising main functional clusters, Table S3: List of kinases and other proteins precipitated with MIBs, Table S4: List of differentially phosphorylated peptides, Figure S1: RH1 treatment affects breast cancer cell cycle, Figure S2. AKT phosphorylation is increased in RH1-resistant cells, Figure S3: Whole western blots.
