# Supplementary material for: Proteomic Analysis of Breast Cancer Resistance to the Anticancer Drug RH1 Reveals the Importance of Cancer Stem Cells
Source: Cancers (Basel). 2019 Jul 11;11(7):972. doi: 10.3390/cancers11070972 (PMC6678540; doi:10.3390/cancers11070972)
Supplement: Supplementary file 1 [file cancers-11-00972-s001.zip › Supplementary Figure S1.pdf]

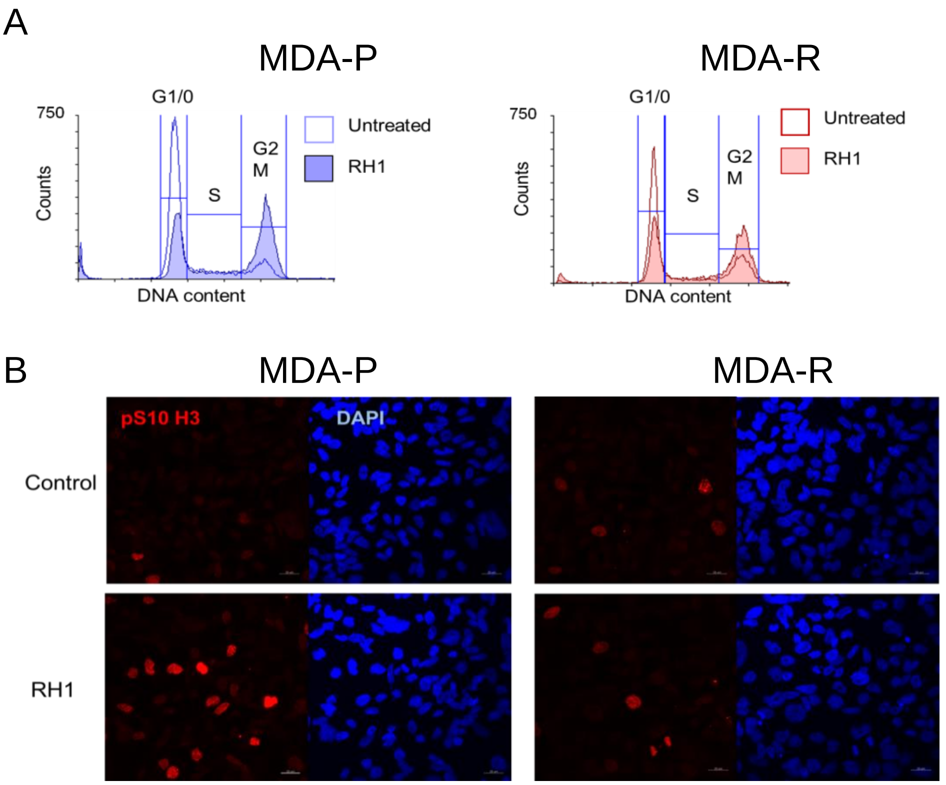

Figure S1. RH1 treatment affects breast cancer cell cycle.
MDA-P and MDA-R cells were untreated or 2 h pulse treated with 10 µM RH1 and then incubated for 1 day until flow cytometry cell cycle analysis or confocal microscopy. A. DNA content flow cytometry analysis after staining with propidium iodide. B. Representative confocal microscopy images of cells stained with antibody against pS10 H3 (red channel) and DAPI (blue channel) untreated (Control) or the drug pulse treated (RH1) cells. Scale bar: 20 µM.
