# Supplementary material for: Proteomic Analysis of Breast Cancer Resistance to the Anticancer Drug RH1 Reveals the Importance of Cancer Stem Cells
Source: Cancers (Basel). 2019 Jul 11;11(7):972. doi: 10.3390/cancers11070972 (PMC6678540; doi:10.3390/cancers11070972)
Supplement: Supplementary file 1 [file cancers-11-00972-s001.zip › Supplementary Figure S2.pdf]

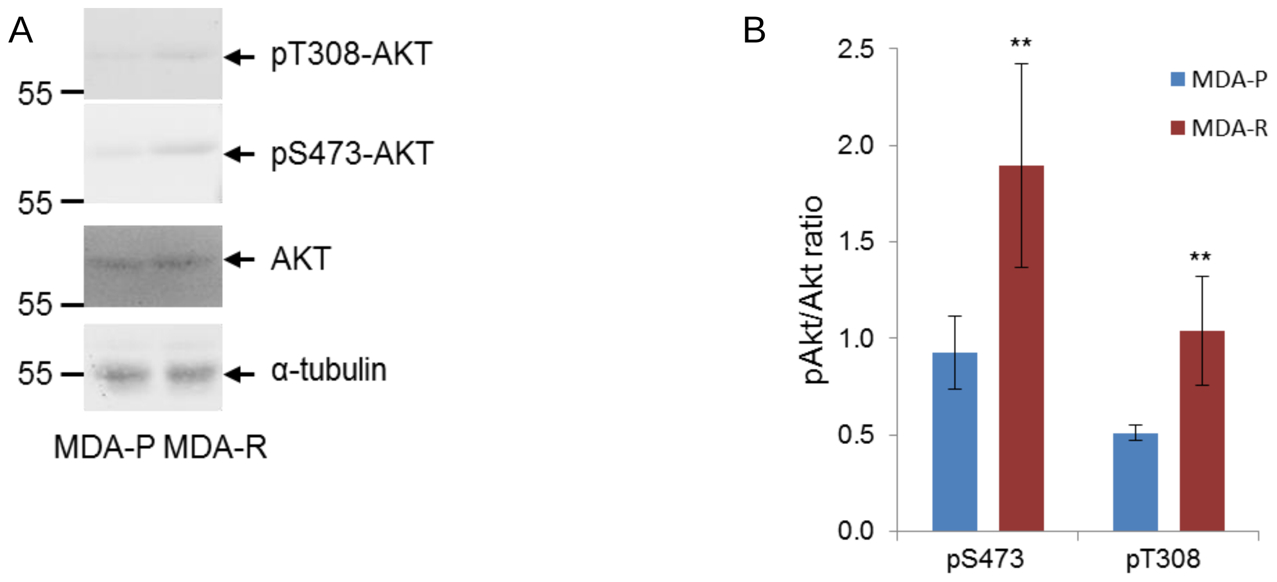

Figure S2. AKT phosphorylation is increased in RH1-resistant cells. A. Western blot analysis showing the level of phosphorylated AKT in MDA-P and MDA-R cells. α-tubulin is shown as loading control. B. Densitometric analysis of pAKT and AKT Western blot. Bars are ± SD, significant difference is marked by asterisks: ** - $P<0.05$, t-test, $n=4$.
