# Supplementary material for: Proteomic Analysis of Breast Cancer Resistance to the Anticancer Drug RH1 Reveals the Importance of Cancer Stem Cells
Source: Cancers (Basel). 2019 Jul 11;11(7):972. doi: 10.3390/cancers11070972 (PMC6678540; doi:10.3390/cancers11070972)
Supplement: Supplementary file 1 [file cancers-11-00972-s001.zip › Supplementary Figure S3.pdf]

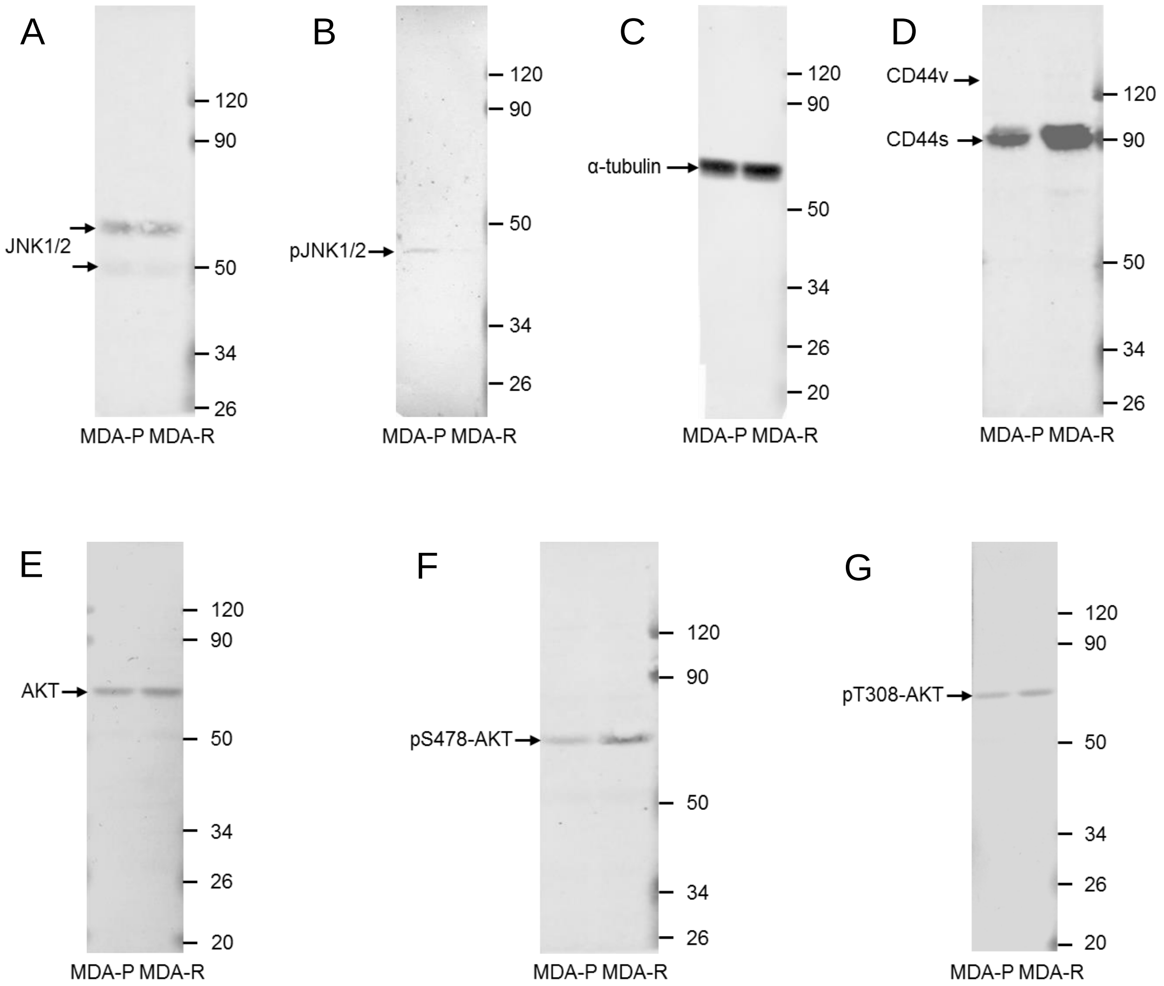

Figure S3. Whole Western blots for (A) JNK, (B) pJNK, (C) α-tubulin, (D) CD44, (E) AKT, (F) pS478-AKT, and (G) pT308-Akt antibodies.
